# The signature of long-standing balancing selection at the human defensin β-1 promoter

**DOI:** 10.1186/gb-2008-9-9-r143

**Published:** 2008-09-25

**Authors:** Rachele Cagliani, Matteo Fumagalli, Stefania Riva, Uberto Pozzoli, Giacomo P Comi, Giorgia Menozzi, Nereo Bresolin, Manuela Sironi

**Affiliations:** 1 Scientific Institute IRCCS E. Medea, Bioinformatic Lab, Via don L. Monza 20, 23842 Bosisio Parini (LC), Italy; 2 Bioengineering Department, Politecnico di Milano, Pzza L. da Vinci, 32, 20133 Milan, Italy; 3 Dino Ferrari Centre, Department of Neurological Sciences, University of Milan, IRCCS Ospedale Maggiore Policlinico, Mangiagalli and Regina Elena Foundation, Via F. Sforza 35, 20100 Milan, Italy

## Abstract

**Background:**

Defensins, small endogenous peptides with antimicrobial activity, are pivotal components of the innate immune response. A large cluster of defensin genes is located on human chromosome 8p; among them the beta defensin 1 (*DEFB1*) promoterhas been extensively studied since discovery that specific polymorphisms and haplotypes associate with asthma and atopy, susceptibility to severe sepsis, as well as HIV and *Candida *infection predisposition.

**Results:**

Here, we characterize the sequence variation and haplotype structure of the *DEFB1 *promoter region in six human populations. In all of them, we observed high levels of nucleotide variation, an excess of intermediate-frequency alleles, reduced population differentiation and a genealogy with common haplotypes separated by deep branches. Indeed, a significant departure from the expectation of evolutionary neutrality was observed in all populations and the possibility that this is due to demographic history alone was ruled out. Also, we verified that the selection signature is restricted to the promoter region and not due to a linked balanced polymorphism. A phylogeny-based estimation indicated that the two major haplotype clades separated around 4.5 million years ago, approximately the time when the human and chimpanzee lineages split.

**Conclusion:**

Altogether, these features represent strong molecular signatures of long-term balancing selection, a process that is thought to be extremely rare outside major histocompatibility complex genes. Our data indicate that the *DEFB1 *promoter region carries functional variants and support previous hypotheses whereby alleles predisposing to atopic disorders are widespread in modern societies because they conferred resistance to pathogens in ancient settings.

## Abstract 2

Analysis of the human beta defensin 1 promoter region in six human populations reveals a signature of balancing selection.

## Background

Defensins comprise a large family of small endogenous peptides with antimicrobial activity against a wide range of microorganisms [1,2]. Although initially regarded as pivotal components of the innate immune system, recent evidence has indicated that defensins also play roles in the recruitment of adaptive immune cells [3] and in promoting antigen-specific immune responses [4].

In humans two defensin subfamilies have been described (α and β), the structural difference residing in the linear spacing and pairing of their six conserved cysteine residues. While α-defensins are expressed by neutrophils and intestinal Paneth cells, β-defensins are mainly produced by epithelia [5].

In mammals, defensins represent large multigene families and a major defensin cluster localizes to human chromosome 8p22-23, where several α- and β-defensin genes are located. Recent evidence [6] has indicated that β-defensin genes on chromosome 8p originated by successive rounds of duplication followed by a complex evolutionary history involving both negative and positive selection with variable pressures among mammalian lineages [7]. Given the relevance of defensins in antimicrobial response and the conundrum whereby increased protein sequence diversity in the immune system enhances the spectrum of pathogen recognition, defensin coding exons have attracted much more interest in evolutionary studies compared to noncoding sequences. Yet, growing evidence suggests that 5' *cis *regulatory regions of genes such as *CCR5 *[8], *HLA-G *[9], *HLA-DQA1 *[10] and *HLA-DPA1/DPB1 *[11] have been subjected to balancing selection during recent primate history.

Among defensins, the human β-defensin 1 (*DEFB1 *[OMIM *602056]) promoter has been extensively studied since specific polymorphisms and haplotypes of it have been associated with asthma and atopy [12], susceptibility to severe sepsis [13], as well as HIV [14,15] and *Candida *[16] infection predisposition. Moreover, recent evidence [17] has indicated that reduced expression of *DEFB1 *is found in a high percentage of renal and prostate cancers, therefore suggesting that *DEFB1 *acts as a tumor suppressor gene. These findings, together with the demonstrated functional significance of polymorphisms within *DEFB1 *5' regulatory sequence, indicate that this region might represent a target of natural selection.

## Results

### Nucleotide diversity at the *DEFB1 *promoter region

**Figure 1:**
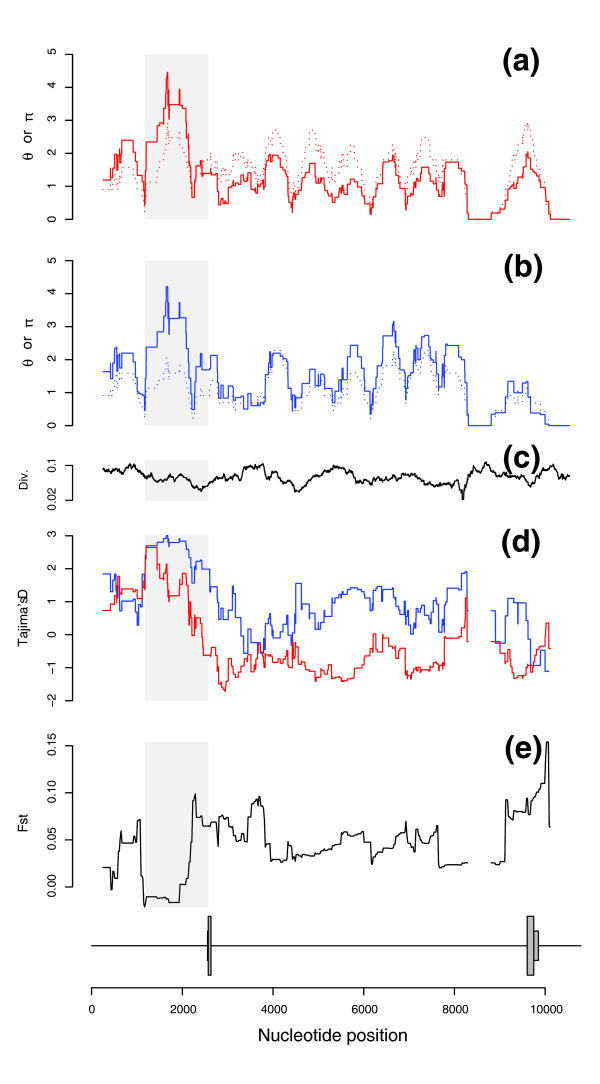
Sliding window analysis along the *DEFB1 *gene sequence. **(a-c) **Analysis of π (solid line) and θ_W _(hatched line) is shown for AA (a, red) and EA (b, blue) together with human-macaque divergence (c). **(d) **Tajima's D for AA (red) and EA (blue). **(e) **Population differentiation between AA and EA as quantified by F_ST_. In all cases, windows of 500 bp with a step of 2 bp were used. The *DEFB1 *gene structure is also shown and the shaded box denotes the region we analyzed.

We sequenced the 1,400 bp region immediately upstream of the *DEFB1 *translation start site (Figure 1) in 83 individuals with different ethnic origins (Yoruba from Nigeria [18] (YRI), Asians (AS), South American Indians (SAI), Australian Aborigines (AUA)); additional data derived from full gene resequencing of 47 subjects (24 African Americans (AA) and 23 European Americans (EA)) were retrieved from the Innate Immunity PGA (IIPGA) web site [19]. A total of 27 single nucleotide polymorphism (SNPs) were identified and haplotypes (Additional data file 1) were inferred using PHASE [20,21]. The analyzed region encompasses all polymorphic variants previously shown to modulate *DEFB1 *expression levels. As a control for the AA and EA populations, data for 20 promoter regions were retrieved for 20 genes in the IIPGA. In particular, the 2 kb upstream of the translation initiation site of other innate immunity genes genotyped for AA and EA were retrieved only if the initial ATG was located in the first exon (as it is for *DEFB1*) and if it could be unequivocally identified. Also, promoter regions were discarded if located in recombination hotspots or in resequencing gaps. A total of 20 promoter regions finally constituted the control dataset. Data concerning the number of segregating sites and nucleotide diversity at the *DEFB1 *promoter region are summarized in Table 1 and indicate that both θ_W _[22] and π [23] are definitely higher for *DEFB1 *compared to maximum values calculated for IIPGA gene promoters.

**Table 1:** Summary statistics of the *DEFB1 *promoter region

		Population					
		
		AA	EA	YRI	AS	SAI	AUA
N^a^		48	46	44	50	48	24
S^b^		22	16	23	17	16	17
							
θ_W _(× 10^-4^)	*DEFB1*	35.41	26.00	37.71	27.11	25.75	32.52
	IIPGA^c^	15.77	14.79	NA	NA	NA	NA
							
π (× 10^-4^)	*DEFB1*	53.73	53.28	45.85	54.96	23.037	51.84
	IIPGA^c^	19.20	17.04	NA	NA	NA	NA
							
Tajima's D	*DEFB1*	1.68^f^	3.29^e^	0.71	3.21^e^	-0.33	2.13^f^
	IIPGA^c^	1.25	1.24	NA	NA	NA	NA
	NIEHS (5 kb)^d^	0.99	0.99	0.93	>0.99	NA	NA
	*p*^g^	0.011	0.0001	0.092	0.0003	NA	NA
							
Fu and Li's D*	*DEFB1*	1.38^f^	1.59^f^	1.085	1.62^e^	1.60^f^	1.23
	IIPGA^c^	1.35	1.36	NA	NA	NA	NA
	NIEHS (5 kb)^d^	>0.99	0.98	0.97	>0.99	NA	NA
	*p*^g^	0.0058	0.0001	0.069	<0.0001	NA	NA
							
Fu and Li's F*	*DEFB1*	1.76^f^	2.56^e^	1.13	2.56^e^	1.12	1.76^f^
	IIPGA^c^	1.46	1.14	NA	NA	NA	NA
	NIEHS (5 kb)^d^	>0.99	0.99	0.96	>0.99	NA	NA
	*p*^g^	0.0031	<0.0001	0.045	<0.0001	NA	NA

^a^Sample size. ^b^Number of segregating sites. ^c^Maximum values for 20 IIPGA gene promoters. ^d^Percentile rank relative to the distribution of 5 kb regions deriving from NIEHS genes. ^e^*p*-value (standard neutral model) <0.01. ^f^*p*-value (standard neutral model) <0.05. ^g^*p*-values obtained by applying a calibrated population genetics model, as described in the text. NA, not available.

We excluded that the high degree of polymorphisms at the *DEFB1 *promoter is due to non-allelic gene conversion with other paralogous defensin genes on chromosome 8 by applying Sawyer's gene conversion algorithm [24].

### Neutrality tests

Under neutral evolution, the amount of within-species diversity is predicted to correlate with levels of between-species divergence, since both depend on the neutral mutation rate [25]. The HKA test [26] is commonly used to verify whether this expectation is verified. We performed both pairwise and maximum-likelihood (MLHKA) [27] tests with *Rhesus macaque *as an outgroup (instead of chimpanzee) so that greater divergence time results in more fixed differences and improves power to detect selection. For pairwise HKA tests we compared polymorphism and divergence level at the promoter region of *DEFB1 *with the 20 IIPGA genes; we consider these comparisons to be well-suited since lower sequence conservation and faster evolutionary rates are though to be a widespread feature of immune response genes [28,29]. Since IIPGA data refer to AA and EA, only these populations were used in the comparison; pairwise HKA tests (Table 2) yielded significant results (*p *< 0.05) in 11 out of 20 cases (with 5 additional tests yielding a *p *< 0.10), suggesting increased diversity at the *DEFB1 *promoter compared to most loci. For further confirmation, we performed a MLHKA test by comparing the *DEFB1 *5' region to all 20 promoter regions: a significant result was obtained (k = 3.31, *p *= 0.0018).

**Table 2:** Pairwise HKA tests

	Intraspecific polymorphisms			Interspecific divergence		
			
Gene	Sample size	Segregating sites	Silent sites	Differences	Sites	HKA *p*-value
*DEFB1*	94	22	1,400	79	1,313	-
*ADAM19*	94	12	2,000	99	1,904	0.11
*CCL2*	94	7	2,000	114	1,989	0.010
*LMAN1*	94	8	2,000	107	1,887	0.018
*LY86*	94	17	2,000	133	1,964	0.13
*PTGDR*	94	15	2,000	108	1,981	0.20
*TGFA*	94	8	2,000	83	1,974	0.074
*TNFRSF18*	94	14	2,000	184	1,602	0.0014
*CCL11*	94	13	2,000	137	1,967	0.038
*CCL5*	94	7	2,000	104	1,726	0.0077
*EGF*	94	10	2,000	96	1,851	0.061
*EGFR*	94	11	2,000	117	1,986	0.047
*IL17E*	94	10	2,000	101	1,978	0.066
*IL17F*	94	14	2,000	87	1,962	0.32
*IRAK3*	94	5	2,000	152	1,952	0.00032
*IL18R1*	94	12	2,000	103	1,901	0.095
*IL23A*	94	3	2,000	81	1,727	0.0030
*MEFV*	94	7	2,000	116	1,941	0.0081
*TGFB2*	94	7	2,000	75	1,960	0.073
*TGFBR1*	94	1	2,000	83	1,991	0.0011
*TLR4*	94	6	2,000	140	1,985	0.0014

Another expectation for neutrally evolving genes is that values of θ_W _and π are roughly equal; this is the case for the maximum values of innate immunity gene promoters but not for *DEFB1*, which shows greater π than θ_W_, a finding consistent with an excess of intermediate frequency variants as a result of balancing selection [30]. The statistics Tajima's D [31] and Fu and Li's D* and F* [32] are commonly used to evaluate the difference between θ_W _and π and, therefore, to test departure from neutrality. As shown in Table 1, significantly positive values for the *DEFB1 *promoter of one or more statistics were obtained for all analyzed populations.

It should be noted that population history, in addition to selective processes, is known [31] to affect frequency spectra and, therefore, all related statistics such as Tajima's D and Fu and Li's D* and F*. In particular, positive values of the statistics are expected under a scenario of population contraction, while negative values are consistent with an increase in population size [31,33]. We performed all tests under the standard assumption of constant population size, which is unrealistic for human populations. Still, this approach is conservative when applied to African populations since they are thought to have undergone moderate but uninterrupted population expansion [34]; in the case of non-African populations the effects of demography are more difficult to disentangle from balancing selection signatures since bottlenecks possibly occurred following migration out of Africa [34]. One possibility to circumvent this problem is to exploit the fact that selection acts on a single locus while demography affects the whole genome. As shown in Table 1, Tajima's D, as well as Fu and Li's F* and D*, displays far higher values in the case of *DEFB1 *compared to the maximum values of innate immunity gene promoters in EA. In order to obtain a more extensive comparison, by including YRI and subjects of Asiatic ancestry we retrieved information concerning 231 genes resequenced in AA, EA, AS and YRI from the NIEHS SNPs Program (NIEHS panel 2) [35]. In particular, for each gene a 5 kb region was randomly selected; the only requirement was that it did not contain any long (>500 bp) resequencing gaps, and if the gene did not fulfill this requirement it was discarded, as were 5 kb regions displaying less than six SNPs. The number of analyzed regions for AA, YRI, EA and AS were 209, 203, 177 and 172, respectively. We calculated the percentile rank of *DEFB1 *values in the distributions of Tajima's D and Fu and Li's F* and D* for this set of loci. In analogy to the results obtained above, values for *DEFB1 *ranked above the 95th percentile in all populations (except for Tajima's D in YRI, which ranked 93rd). It is worth mentioning that, as already noticed by other authors [36], resequenced genes in SNP discovery programs probably represent a sample biased toward non-neutrally evolving loci (in the case of the NIEHS SNPs Program, genes are selected on the basis of their having a role in organism-environment interactions), making comparison with their distribution a conservative test.

A second possibility to disentangle the effect of demographic history from selection is to apply calibrated population genetics models. In particular, one such model that has been proposed recently, *cosi *[37], is based on the ability to generate realistic data rather than relying on inference about population histories. We performed coalescent simulations using the *cosi *package [37] and its best-fit population parameters for YRI, AA, EA and AS. Data are reported in Table 1 and indicate that for Tajima's D, as well as for Fu and Li's D* and F*, application of a calibrated model allows rejection of neutrality for the four populations at the *DEFB1 *promoter region.

Population genetic differentiation, quantified by F_ST _[38], can also be used to detect the signature of balancing selection. In particular, lower F_ST _values are expected at loci under balancing selection compared to neutrally evolving ones [39,40]. F_ST _among AA, EA and AS was 0.0057, much lower than the genome average of 0.123 [40] and not significantly different from 0 (*p *= 0.25).

We next wished to verify that the evolution of the *DEFB1 *promoter is not influenced by the presence of a linked balanced polymorphism within, for example, the gene coding region. We exploited the availability of full resequencing data for the whole gene and calculated human-macaque divergence, nucleotide diversity, Tajima's D and F_ST _in sliding windows for AA and EA. As shown in Figure 1, while inter-specific divergence is quite homogeneous along *DEFB1*, a peak in nucleotide diversity (expecially π) is observed at the promoter; consistently, in both AA and EA, the same region displays the maximum Tajima's D value and the minimum F_ST_, with no other region showing evidence suggestive of balancing selection.

It should be noted that several defensin genes on 8p23.1, but not *DEFB1*, exhibit copy number variation (CNV) in humans [41]; a more recent [42] genome-wide analysis of CNVs indicated that the 5' gene region of *DEFB1 *might be encompassed by a CNV, although the authors indicate that, since the breakpoints are difficult to establish, involved loci might flank rather than be encompassed by the CNVs. The authors studied HapMap subjects and reported a frequency for the CNV ranging from 6% to 14% in different populations. Since our YRI samples comprise a subset of HapMap YRI subjects, we checked whether any of them were reported to display a CNV in this region: two subject were retrieved, accounting for one gain and one loss. Electropherograms of these two subjects (as well as all other subjects in this study) revealed no evidence of unbalanced peaks at heterozygous SNPs and their removal from the sample did not affect the results for YRI. Previous [43] work had studied CNVs in the defensin cluster on chromosome 8 using real-time PCR assays and found that 24 American subjects with different ethnic origin had 2 copies of *DEFB1*. Taking these observations together, we consider that either *DEFB1 *lies outside the CNV or, in any case, that CNVs encompassing *DEFB1 *are very rare and do not affect the results reported here.

### Haplotype analysis

**Figure 2:**
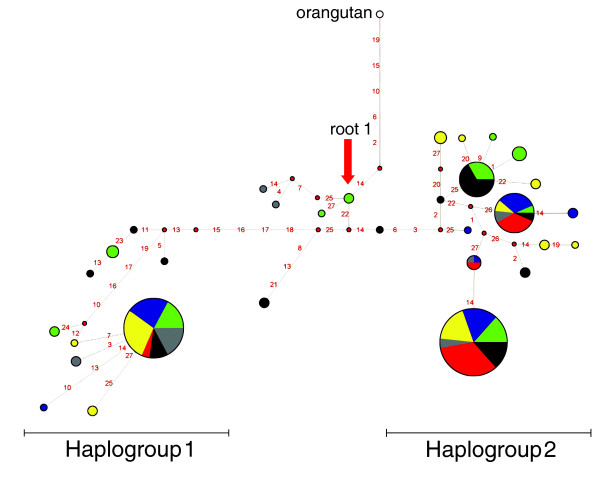
Genealogy of *DEFB1 *haplotypes reconstructed through a median-joining network. Each node represents a different haplotype, with the size of the circle proportional to the haplotype frequency. Also, circles are color-coded according to population (green, AA; black, YRI; blue, EA; yellow, AS; red, SAI; gray, AUA). The red arrow indicates root 1 (see text). Nucleotide differences between haplotypes are indicated on the branches of the network. The orangutan sequence is also shown.

One effect of balancing selection is to preserve two or more lineages over an extended period of time, resulting in clades separated by long branch lengths. To examine the genealogy of *DEFB1 *promoter haplotypes, we built a median-joining network. The topology of this network (Figure 2) is unambiguous with no reticulations, a pattern consistent with the low level of recombination observed in this gene region (not shown). Two major clades (haplogroups 1 and 2) separated by long branch lengths are evident, each containing one common haplotype. We next wished to estimate the time to the most recent common ancestor (TMRCA) of the two haplotype clades, applying a phylogeny-based method [44] based on the measure ρ, the average distance of descendant haplotypes from a specified root. By using root 1 (Figure 2), ρ was equal to 9.45 so that, with a mutation rate based on 21 fixed differences between chimpanzee and humans and a separation time of 5 million years ago, we estimated a TMRCA of 4,489,791 years (standard deviation ±1,018,128).

### Comparison with other primates

In order to gain further insight into the evolutionary history of the *DEFB1 *promoter region, we resequenced those from three chimpanzees and one orangutan. These samples were obtained from the European Collection of Cell Cultures and the *Pongo *sequence was used in the median-joining network in order to root the phylogeny (Figure 2). A total of 5 polymorphic sites were identified in chimpanzees; one of them (-913 C/T in the human sequence) was shared with humans and, therefore, represents a *trans*-specific polymorphism. *Trans*-specific polymorphisms are an effect of long-term balancing selection, while they are highly unlikely under neutrality. Indeed, a neutral polymorphism is expected to persist for 4N_e _generations (where N_e _is the effective population size, estimated to be around 10,000 for humans) [45] and, therefore, the probability of observing a polymorphism shared between humans and chimpanzees, two species that diverged about 5 million years ago (around 20N_e _generations), is extremely low [46,47]. Although the identification of a human/chimpanzee *trans*-specific SNP is consistent with the estimated TMRCA of the haplotype clusters (suggesting that balancing selection was established around the same time when the human and *Pan *lineages split), the possibility exists that the shared SNP is due to a coincidental mutation that occurred after speciation. Indeed, the location of the substitution at a CpG site makes the possibility of a recurrent mutation more likely and, therefore, taking into account the lack of functional data on this SNP, it is difficult to discriminate between the two possibilities.

## Discussion

Haldane's hypothesis [48] as formulated in 1932 posits that infectious diseases have been a major threat to human populations and have, therefore, exerted strong selective pressures throughout human history. As a result, a number of human loci are thought to have evolved in response to such pressures. Up to now, most evolutionary studies have focused on adaptive immunity, yet the ancient innate immune system, with the production of antimicrobial peptides, provides a critical line of defense in vertebrates [5]. Following Haldane's idea, it is conceivable, therefore, that innate immunity genes have undergone similar selective pressures as their adaptive counterparts. Indeed, in analogy to immunoglobulins [49] and major histocompatibility complex (MHC) molecules [50], the paradigm whereby gene duplication followed by rapid divergence has been a powerful adaptive strategy in immune response genes has been verified for defensin loci [6,7,51]. Recent studies [7] demonstrated that, after gene duplication in an ancestral mammalian genome, the mature peptide-coding exons of β-defensins have been subjected to positive selection, while sites within the pre-propeptide region have undergone negative selection in primate lineages.

The data we report add further complexity to the evolutionary history of defensin genes by showing that balancing selection has shaped variability at the promoter region of human *DEFB1*. Indeed, we have documented here that the *DEFB1 *promoter region displays elevated nucleotide diversity, excess of polymorphism to divergence levels and reduced population differentiation. In line with these findings, the analysis of *DEFB1 *haplotypes revealed the presence of two clades separated by long branches approximately dating back to the time when the human and chimpanzee lineages split. Altogether, these features represent strong molecular signatures of long-term balancing selection, a process that is thought to be extremely rare outside MHC genes [47].

β-Defensin 1, the first human β-defensin to be discovered, shows anti-bacterial activity against a wide range of Gram-negative bacteria (for example, *Escherichia coli*, *Pseudomonas aeruginosa*, and *Klebsiella pneumoniae*), as well as different *Candida *species [52-54]. β-defensin 1 is constitutively expressed by most epithelia with higher levels being detectable in kidney, pancreas, the urogenital and respiratory tracts [54-56]. Consistently, targeted disruption of the mouse β-defensin 1 gene resulted in animals deficient in the clearance of *Haemophilus influenzae *from the lung [57] or containing a greater number of bacteria (Staphylococci, in particular) in urine collected from the bladder [58]. Also, *DEFB1 *expression has been demonstrated [59-61] in the human epidermis, gingival epithelium, oral mucosa and saliva, suggesting that it contributes to host defenses in areas exposed to a variety of microbial challenges. Moreover, recent evidences indicated that the protein product of *DEFB1 *is detectable in human milk [62] and the mammary epithelium [63]; in particular, pregnant women display higher levels of β-defensin 1 and concentrations comparable to those observed in milk were effective in killing *E. coli *[62], suggesting that this antimicrobial peptide might have a fundamental role in protecting breast-fed infants from infectious diarrhea and mothers from lactational mastitis [62,63].

The promoter region of *DEFB1 *has recently been subjected to extensive study; in particular, three SNPs have been reported to affect gene expression [17,64], although contrasting results on transcriptional activity have been obtained by different research groups, possibly reflecting either non-trivial interactions among polymorphic alleles at multiple positions or cell-type specific SNP effects [65]. In SNP typing studies, the -20A/-44C/-52G haplotype has been independently associated with protection against severe sepsis [13], susceptibility to asthma and atopy [12] and, in cystic fibrosis patients, with chronic *P. aeruginosa *lung infection [66]. Also, the -44C allele was shown to predispose to HIV [14,15] and *Candida *[16] infection, while an association with HIV infection in Brazilian children was also reported for SNPs -20G and -52A [15]. Although the biological bases for these associations are presently unknown, their description allows interesting speculations concerning the selective pressures possibly shaping nucleotide diversity at the *DEFB1 *promoter region. Sepsis is a leading cause of death in infants and children throughout the world [67]; its incidence and fatal outcome were conceivably higher before the advent of modern sanitation and, therefore, it might have represented a powerful selective force during human history. Indeed, signatures of natural selection have been reported at another human locus, namely *CASP12 *[68], as a possible adaptive response to sepsis. Variants in the *DEFB1 *promoter that protect against sepsis might, therefore, have conferred a selective advantage to carriers, although one or more of these same SNP alleles have been associated with predisposition to candidiasis [16], as well as to susceptibility to HIV and *P. aeruginosa *infection (at least in cystic fibrosis patients) [14,15,66]. In this respect, it is interesting to notice that early hunter-gatherer societies, due to their small population sizes, were likely to support a parasite fauna constituted of pathogens with high transmission rates and inducing little or no immunity [69]. In such a scenario, the role of innate response might have been extremely relevant to ensure protection from infectious agents. The increase in population size that occurred at some time during human history is thought to have allowed maintenance of a different and wider range of pathogen species, including major infectious agents responsible for sepsis. Variable environmental conditions are regarded as a possible explanation underlying the maintenance of balanced polymorphisms [70]; in a simplistic situation whereby a variant (or haplotype) protects against sepsis while predisposing to other infectious agents, changes in pathogen prevalence, with particular reference to microbes leading to fatal sepsis, might modulate the fitness of subjects carrying either allele.

Unfortunately, little information is available concerning the early epidemiological history of our predecessors; indeed, the timing of human population expansion has been matter of debate [71-73] and some uncertainty concerns the time of origin of major human pathogens, for example, tuberculosis [74,75]. Further studies concerning these issues, as well as better understanding of the role of *DEFB1 *polymorphisms, will therefore be required before a direct link can be established between pathogen-driven selective pressure and the maintenance of *DEFB1 *variants.

An additional, non-mutually exclusive possibility to explain the action of balancing selection at the *DEFB1 *promoter implies heterozygote advantage. This phenomenon is deemed responsible for maintenance of polymorphisms at MHC class II promoters [10,76] and is thought to enhance immune response flexibility by modulating allele-specific gene expression in different cell-types [77] and in response to diverse stimuli/cytokines [78]. *DEFB1 *is considered a constitutive defensin, in that, unlike β-defensin 2, it shows limited inducibility by inflammatory stimuli (reviewed in [5]); however, previous reports have indicated that *DEFB1 *shows marked inter-individual variability in expression levels in urine, saliva, gingival epithelium and epidermis [56,59-61]. Similarly, the ability of lipopolysaccharide to induce *DEFB1 *expression varied among the blood samples obtained from 51 healthy individuals [53]. These data, together with the functional data indicating allele-dependent promoter activity in different cell types [64,65], suggest that *DEFB1 *variants might exert different effects in diverse tissues, possibly accounting both for inter-individual variation of expression levels and for maintenance of divergent clades.

It might also be worth mentioning that evidence, albeit preliminary, indicates that *DEFB1 *expression is up-regulated during pregnancy [56,62], suggesting hormone-regulated gene expression. No data have ever been reported concerning the response of different *DEFB1 *promoter haplotypes to hormone treatment; were any difference identified, the adaptive significance of variants increasing expression in human milk, for example, would be evident.

Finally, it might be interesting to note that, given its high expression in urogenital tissues, *DEFB1 *has been regarded as a possible innate defense against sexually transmitted pathogens [56]. In line with this view, induction of an antiviral response in cultured uterine epithelial cells resulted in a six-fold increase in *DEFB1 *expression [79]. Since sexually transmitted diseases are thought to have affected early hominid societies, due to their sustainability in low-density host population [69], these observation might help to explain the ancient origin of *DEFB1 *haplotype clades.

As discussed in the introduction, two recent reports indicated that balancing selection has shaped variability at the promoter region of other loci involved in immune response. In the case of *CCR5*, available evidence indicates that heterozygosity at this gene region delays HIV-1 disease progression [80]. However, as the authors note, the introduction of HIV-1 in human populations is relatively recent and cannot, therefore, account for the maintenance of balanced polymorphisms in the region; therefore, *CCR5 *possibly evolved to respond to older pathogens, providing a clue to the difficult task of inferring the origin of selective pressures exerted by human pathogens over long evolutionary times.

Whatever the reason for the maintenance of a balanced variant, it is interesting to note that variation at *DEFB1 *might fit a previously proposed hypothesis [81] whereby alleles that conferred resistance to pathogens in ancient settings are now associated with susceptibility to atopic disorders; *DEFB1 *haplotypes associated with protection against sepsis seem to predispose to asthma and atopy. A similar link between past selection and present disease predisposition has been suggested [82] in the case of polymorphic variants in the *IL4RA *gene and might help to explain the high prevalence of atopic conditions in modern societies.

## Conclusion

Association studies of *DEFB1 *variants have focused on a small number of SNPs to be genotyped; it is possible, therefore, that additional variants in this gene region play a role in the above described (or still unknown) conditions. In this regard, it is worth mentioning that the availability of full gene resequencing data allowed us to define a specific *DEFB1 *gene region as the target of balancing selection and, therefore, as the location of functional variants. This information might be valuable in future association studies, suggesting that *DEFB1 *promoter SNPs, rather than linked variants, associate with specific phenotypes.

This report represents an example of how population genetics approaches may benefit from association studies by gaining cues about possible selective pressures acting on target gene regions; we hope it also illustrates the possible contribution of evolutionary models to classic SNP-disease association approaches by providing information about the localization of candidate functional variants.

## Materials and methods

### DNA samples and sequencing

Human genomic DNA was obtained from the European Collection of Cell Cultures (Ethnic Diversity DNA Panel plus additional samples for Australian Aborigine derived from HLA defined panels). From the same source we obtained the genomic DNA of three chimpanzees (*Pan troglodytes*) and one orangutan (*Pongo pygmaeus*). Additional DNA samples from South American Indians and Yoruba individuals were derived from the Coriell Institute for Medical Research.

The 1.4 kb region covering the promoter region of *DEFB1 *was PCR amplified (primer sequences are reported in Table 3). PCR products were treated with ExoSAP-IT (USB Corporation, Cleveland, OH, USA), directly sequenced on both strands with a Big Dye Terminator sequencing Kit (v3.1 Applied Biosystems, Monza, Italy) and run on an Applied Biosystems ABI 3130 XL Genetic Analyzer. All sequences were assembled using AutoAssembler version 1.4.0 (Applied Biosystems), inspected manually by two distinct operators, and singletons were re-amplified and resequenced.

**Table 3:** Primer sequences

	Forward primers	Reverse primers
H	DEFB1-F1:CAATCTCACTGCTCCTAGGTC	DEFB1-R1:CAGGAATGACATCCACCCTAC
	DEFB1-F2:CTGCCAGCGGTAGAGTGGC	DEFB1-R2:CTGGTGCCAGCTCCTCCTG
	DEFB1-F3:CTCCAGTGTGAACTGCCTG	DEFB1-R3:CTTGCCTGCTGCCTTCTGC
		
C	DEFB1-C-F1:CAATCTTATTGAACCCACAC	DEFB1-C-R1:CAAGTATTCCTCAGGTTTTC
	DEFB1-C-F2:CTGCCAGGGGTAGAGTGGC	DEFB1-C-R2:CTGGGGCCAGCTCCTCCTG
	DEFB1-C-F3:GGATTCCAGTGTGAACTGCC	DEFB1-R3:CTTGCCTGCTGCCTTCTGC

Primers used for amplification of human (H) and chimpanzee (C) templates.

### Data retrieval and haplotype construction

*DEFB1 *genotype data for American subjects of either African or European descent were retrieved from the IIPGA website [19]. From the same source, we derived resequencing data referring to promoter regions (2 kb upstream of the translation initiation site) of other innate immunity genes genotyped for AA and EA. Promoter regions were not selected if the initial ATG was not located in the first exon (as it is for *DEFB1*) or if it could not be unequivocally identified due to the presence of multiple 5' isoforms, which were identified through manual inspection of UCSC annotation tracks [83]. Also, promoter regions were discarded if located in recombination hotspots (these were manually identified through the UCSC genome annotation tables snpRecombHotspotHapmap and snpRecombHotspotPerlegen [83]) or in resequencing gaps. A total of 20 promoter regions finally constituted the control dataset.

Genotype data for 231 resequenced human genes were derived from the NIEHS SNPs Program web site [35]. In particular, we selected genes that had been resequenced in populations of defined ethnicity, including Asians (NIEHS panel 2).

Haplotypes were inferred using PHASE version 2.1 [20,21], a program for reconstructing haplotypes from unrelated genotype data through a Bayesian statistical method. Haplotypes for AS, AUA, SAI and YRI individuals are available as supporting information (Additional data file 1).

### Statistical analysis

Tajima's D [31], Fu and Li's D* and F* [32] statistics, as well as diversity parameters θ_W _[22] and π [23] were calculated using *libsequence *[84], a C++ class library providing an object-oriented framework for the analysis of molecular population genetic data. Departure from neutrality was tested from coalescent simulations computed with *ms *software [85] fixing the mutation parameter, assuming no intra-locus recombination and a constant population size with 100,000 iterations. Calibrated coalescent simulations were performed using the *cosi *package [37] and its best-fit parameters for YRI, AA, EA and AS populations with 10,000 iterations. The F_ST _statistic [38] estimates genetic differentiation among populations and was calculated as proposed by Hudson *et al*. [86]. Significance was assessed by permuting 10,000 times the haplotype distribution among populations [87].

Pairwise HKA tests were performed using *libsequence*. The maximum-likelihood-ratio HKA test was performed using the MLHKA software [27] with multilocus data of 20 selected IIPGA promoter regions and *Rhesus macaque *(NCBI rheMac2) as an outgroup. In particular, we evaluated the likelihood of the model under two different assumptions: that all loci evolved neutrally and that only the *DEFB1 *promoter region was subjected to natural selection; statistical significance was assessed by a likelihood ratio test. We used a chain length (the number of cycles of the Markov chain) of 500,000 and, as suggested by the authors, we ran the program several times with different seeds to ensure stability of results.

In order to test for gene conversion events, we applied Sawyer's gene conversion algorithm [24] implemented in the GENECONV program. GENECONV assesses significance using two methods: permutations and an approximate *p*-value [88,89]. We performed several tests by varying the mismatch penalty from 0 to larger positive values and using 10,000 permutations. For all these runs and both methods, no pairwise or global *p*-value involving *DEFB1 *was significant, suggesting no inner or outer fragments showing past gene conversion.

The median-joining network to infer haplotype genealogy was constructed using NETWORK 4.2 [44]. The time to the most common ancestor (TMRCA) was estimated using a phylogeny based approach implemented in NETWORK 4.2 using a mutation rate based on 21 fixed differences between chimpanzee and humans in the 1.4 kb *DEFB1 *region.

All calculations were performed in the R environment [90].

## Abbreviations

AA, African American; AS, Asian; AUA, Australian Aborigine; CNV, copy number variation; EA, European American; IIPGA, Innate Immunity PGA; MHC, major histocompatibility complex; SAI, South American Indian; SNP, single nucleotide polymorphism; TMRCA, time to the most recent common ancestor; YRI, Yorubans.

## Authors' contributions

RC and SR performed all resequencing experiments and analyzed the data. MF and GM retrieved genotype data and performed population genetics analyses. MS, MF, RC, GPC and UP analyzed and interpreted the data. NB participated in the study coordination. MS and MF wrote the paper. MS conceived and coordinated the study.

## Additional data files

The following additional data are available. Additional data file 1 is a spreadsheet reporting the *DEFB1 *promoter haplotypes for the following subjects: 22 YRI, 25 AS, 24 SAI and 12 AUA. SNP positions refer to the NCBI Build 36.1 assembly.

## Supplementary Material

Additional data file 1 *DEFB1 *promoter haplotypes are reported for AS, AUA, SAI and YRI. Click here for file
